# Clinical characteristics of non-tuberculous mycobacterial pulmonary infections in Bamako, Mali

**DOI:** 10.1017/S0950268817003090

**Published:** 2018-01-15

**Authors:** B. Kone, Y. S. Sarro, M. Maiga, M. Sanogo, A. M. Somboro, B. Diarra, A. C. G. Togo, N. Coulibaly, B. P. P. Dembele, D. Goita, B. Baya, A. Kone, S. Diabaté, M. A. Polis, M. Belson, S. Dao, S. Orsega, C. J. Achenbach, R. L. Murphy, S. Diallo, S. Siddiqui

**Affiliations:** 1 University of Sciences, Techniques and Technologies of Bamako (USTTB), Bamako, Mali; 2 Northwestern University, Chicago, Illinois, USA; 3 National Institute of Allergic and Infectious Diseases, DCR/CCRB, Bethesda, Maryland, USA

**Keywords:** Bamako, clinical characteristics, non-tuberculous mycobacteria, tuberculosis

## Abstract

The global spread of non-tuberculous mycobacteria (NTM) may be due to HIV/AIDS and other environmental factors. The symptoms of NTM and tuberculosis (TB) disease are indistinguishable, but their treatments are different. Lack of research on the epidemiology of NTM infections has led to underestimation of its prevalence within TB endemic countries. This study was designed to determine the prevalence and clinical characteristics of pulmonary NTM in Bamako. A cross-sectional study which include 439 suspected cases of pulmonary TB. From 2006 to 2013 a total of 332 (76%) were confirmed to have sputum culture positive for mycobacteria. The prevalence of NTM infection was 9.3% of our study population and 12.3% of culture positive patients. The seroprevalence of HIV in NTM group was 17.1%. Patients who weighed <55 kg and had TB symptoms other than cough were also significantly more likely to have disease due to NTM as compared to those with TB disease who were significantly more likely to have cough and weigh more than 55 kg (OR 0.05 (CI 0.02–0.13) and OR 0.32 (CI 0.11–0.93) respectively). NTM disease burden in Bamako was substantial and diagnostic algorithms for pulmonary disease in TB endemic countries should consider the impact of NTM.

## Introduction

Non-tuberculous mycobacteria (NTM) are a major cause of opportunistic infection in immunocompromised hosts. NTM include over 125 different species of mycobacteria ubiquitous in the environment [1] and cause morbidity worldwide [2]. NTM species implicated in human disease can produce non-specific symptoms ranging from asymptomatic to destructive or even fatal disease [1]. In cases where symptoms and signs occur, they are often indistinguishable clinically and radiographically from those caused by *Mycobacterium tuberculosis* (*TB*) [3]. Consequently, many NTM-infected patients in TB endemic countries, such as Mali, receive unnecessary long treatment with anti-TB drugs that are not only expensive but also toxic [4]. In Mali, during a study conducted at SEREFO laboratory in Bamako, we found that 18% of TB treatment failure patients had NTM disease [4]. In TB endemic countries, NTM disease was considered to be negligible until more recent reports indicated the impact they may have in the management of patients with TB-like symptoms [4–7]. In addition, many recent reports revealed that NTM is increasing worldwide [8]. Reasons for this increase is unclear but environmental changes, availability of better detection tools and greater disease awareness are likely contributing factors [8, 9].

In high-income countries, the prevalence of NTM is estimated to be between 1 and 1.8 cases per 100 000 [10] with infections by *Mycobacterium avium* complex (MAC) as the most common [11]. In the US, the Center for Disease Control and Prevention reported that MAC is the cause of 75% of NTM-related respiratory infections [5, 12], and MAC was followed by *Mycobacterium fortuitum* and *Mycobacterium kansasii* [5]. In Denmark, the annual incidence rate of NTM disease is 1.20/10^5^ with a majority of infections also due to MAC [9]. In Cambodia, a low-middle income country, the prevalence of NTM disease was considered high at 10.8% of patients with presumptive TB in a recent study [13].

Mali, a West African country, with an estimated population of 17 million, had a prevalence of TB of 90/100 000 and general HIV seroprevalence of 1.1% in 2014 [14–16]. Sputum smear microscopy is the only tool available to diagnose TB in most areas and physicians are unable to differentiate between TB and NTM pulmonary disease. The extent to which NTM contributes to TB treatment failure and development of drug resistance is uncertain. Thus, we sought to evaluate the epidemiology of NTM disease in Mali by examining the prevalence of NTM disease and its associated factors in patients with suspected pulmonary TB at the Pneumology Department of the Point-G University Teaching Hospital of Bamako (PGUTH).

## Methods

### Study type and population

We performed a cross-sectional study conducted from January 2006 to December 2013 in which we included patients referred for presumptive TB at the PGUTH. We enrolled all patients suspected of having TB who were 14 years or older with persistent cough for over 15 days, fever, night sweats and/or weight loss. The city of Bamako is administratively divided into six municipalities with each having a local health referral center capable of diagnosing and treating TB patients. These centers refer all TB treatment failure patients to the PGUTH where our study took place. The study participants were referred from the six health referral centers of Bamako. The PGUTH receives 50–100 treatment failure cases each year.

### Laboratory testing

We performed laboratory testing at the SEREFO Biosafety Level-3 Laboratory (BSL-3) facility affiliated with the University of Sciences, Techniques, and Technologies of Bamako (USTTB) and the US National Institutes of Health/National Institute of Allergy and Infectious Diseases (NIH/NIAID). We performed sputum smear stained with Auramine Rhodamine and sputum culture (MGIT™ and 7H11) before molecular identification of mycobacterial species as previously described [4]. In brief, nucleic acid probes (AccuProbe, Gen-Probe, San Diego CA, USA) for *M. tuberculosis* complex (MTBc), MAC, *Mycobacterium gordonae* and *M. kansasii* were used for initial identification and sequencing was performed as needed using published protocols [17, 18].

For HIV status, we performed a rapid diagnostic test by Determine^®^ (Determine^®^ HIV-1/2, Abbott Laboratories, Matsudo Shi, Chiba, Japan), followed by Genscreen™ ULTRA HIV Ag-Ab (Genscreen, HIV-1/2 Version 2 Assay, Bio-Rad Laboratories, Marnes – La Coquette, France). Western blot (New Lav Blot I and II, Bio-Rad Laboratories, Marnes – La Coquette, France) was used for confirmatory testing of all positives.

### Clinical examination

We conducted a standardised interview, performed physical examination and collected three consecutive early morning sputum samples. Blood samples were also collected for HIV testing and haematological parameters. We completed a case report form for each participant to capture socio-demographic data (profession, sex, age, address, level of education, HIV status and others), clinical symptoms, laboratory results and medications.

### Data analysis

Fisher's test or *χ*^2^ test was used for the comparison of frequencies and *t* test for comparison of means. Multivariate logistic regression was performed with NTM (yes/no) as the dependent variable. In univariate and multivariate models, we assessed the following risk factors for NTM: gender, age, weight loss, fever, heart rate, rales crackling, night sweat, cough, leucocytes and haemoglobin. We considered *P* value <0.05 of comparisons as statistically significant and significant factors in univariate analyses were included in multivariate models. Multivariate models also included age and sex regardless of *P* value since they were known risk factors for NTM disease. EPI-info version 7.0.9 and SAS version-9 were utilised to conduct these statistical analyses.

### Ethical considerations

The study protocol was approved by the Ethics Committee of the University of Bamako and the Institutional Review Board (IRB) of the US National Institutes of Health. All participants provided informed consent and signed a study-specific consent form prior to enrolment.

## Results

### Study population and infections’ prevalence

**Fig. 1.**
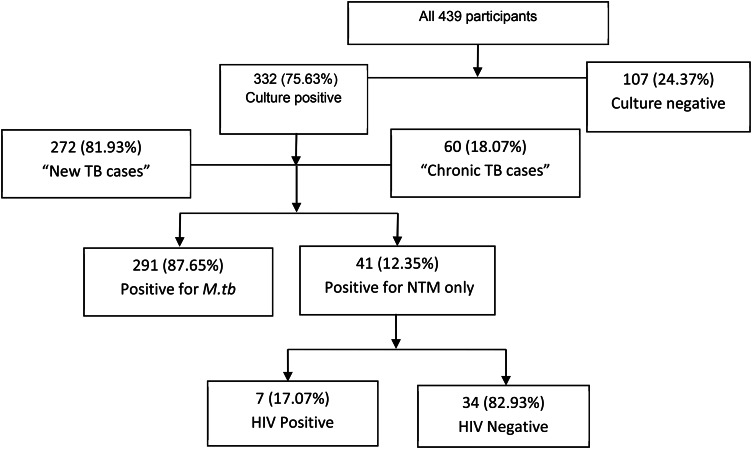
Descriptive groups of the study.

A total of 439 patients were included (Fig. 1) in our analysis. Of those included, 77% (336/439) were male, mean age was 35.8 years (14–83) and 87.24% (383/439) resided in the Bamako District with 132 participants (30.07%) from the Commune VI part of this district. There were no significant differences in educational level between the groups.

**Fig. 2.**
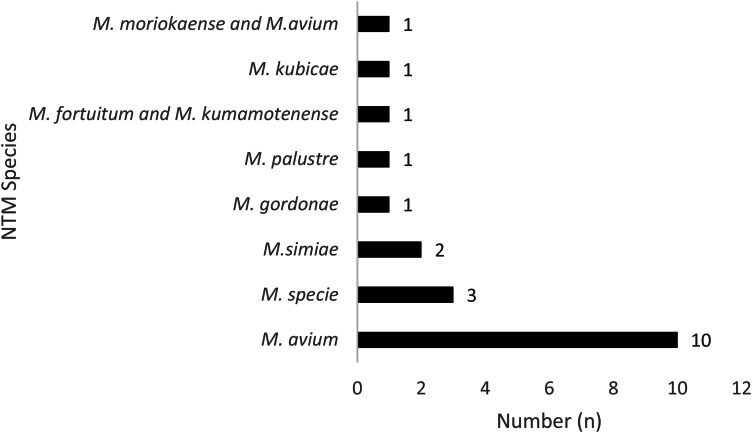
Distribution of the 20 non-tuberculosis mycobacteria species in Bamako, Mali.

After sputum analysis, we found that 9.34% (41/439) were NTM, 66.29% (291/439) were *M. tuberculosis* complex and 24.37% (107/439) were sputum culture negative (Fig. 1). Among those with sputum culture positive, NTM was identified in 12.34% (41/332) and *M. tuberculosis* complex in 87.66% (291/332). The 41 NTM were identified morphologically and by fluorescent acid-fast microscopy with solid medium cultures, to be mycobacteria that are not *M. tuberculosis* (negative for MTBc Gen-Probe). Twenty of the 41 NTM were further formally specified (the type of NTM) using other available probes or 16S sequencing (Fig. 2). Of these NTM cases, 41.46% (17/41) were new TB suspects and 58.54% (24/41) were identified as failing TB treatment. Male sex was more common in both the *M. tuberculosis* complex group with 76.29% (222/291) and the NTM group with 80.49% (33/41) (Table 1). The most affected age category was 14–30 year olds with 46.74% (136/291) in *M. tuberculosis* complex group and 30–45 year olds with 46.34% (19/41) in the NTM group. Of note, when both TB and NTM were identified in the same sputum sample, we categorised the patient as having TB disease with NTM colonisation as per American Thoracic Society (ATS) definition [5].

**Table 1.** Socio-demographic and clinical characteristics of tuberculous and non-tuberculous mycobacteria (NTM)-infected patients, from 2006 to 2013 in Bamako, Mali

	*Mycobacterium tuberculosis* *N* = 291 (87.65%)	NTM *N* = 41 (12.34%)
Age range		
14–30	136 (46.74)	7 (17.07)
30–45	94 (32.30)	19 (46.34)
45–60	49 (16.84)	9 (21.95)
60–75	9 (3.09)	5 (12.20)
>75	3 (1.03)	1 (2.44)
Gender		
Female	69 (23.71)	8 (19.51)
Male	222 (76.29)	33 (80.49)
Symptoms		
Bacillus impregnation syndrome^a^	158 (54.29)	19 (46.34)
Cough	46 (15.81)	17 (41.46)
Thoracic pain	43 (14,78)	1 (2.34)
Dyspnoea	34 (11.68)	1 (2.34)
Diarrhoea	10 (3.44)	3 (7.31)

a Bacillus impregnation syndrome: group of symptoms made of asthenia, anorexia fever, night sweats and weight loss.

### Symptoms associated with infections

Among those who were culture positive for *M. tuberculosis* complex, we observed symptoms of bacillus impregnation in 54.29% (158/291), thoracic pain in 14.78% (43/291) and cough in 15.81% (46/291) of the participants (Table 1). However, in the NTM disease group, we observed symptoms of bacillus impregnation in 46.34% (19/41), thoracic pain in 2.34% (1/41) and cough in 12.19% (17/41). The bacillus impregnation syndrome is a group of symptoms made of asthenia, anorexia fever, night sweats and weight loss [19]. Overall, body weight more than 55 kg at time of referral to the study was less likely to be associated with pulmonary NTM disease (OR = 0.53).

There were no statistically significant differences in haematological parameters between those with TB and NTM disease (data not shown).

### NTM disease

At inclusion, the mean weight of those with NTM disease was 51.05 ± 8.7 kg and the mean heart rate was 96.53 ± 17.63 beats per minute. Furthermore, of the 41 patients who met ATS criteria for NTM disease, weight was 51 ± 8.79 kg, the mean of body temperature was 36.93 ± 0.90 °C, the mean heart rate 108 ± 29.75 beats per minute and the mean respiratory rate was 29.75 ± 10.09 breaths per minute.

In addition, 17.07% (7/41) of those with NTM disease were HIV seropositive. Of the NTM specified, 50% (10/20) were identified as having *M. avium*, 15% *Mycobacterium* species (3/20) and 10% (2/20) *Mycobacterium simiae* (Fig. 2). Of the NTM/HIV co-infected participants, 71.43% (5/7) were living in the Commune VI of Bamako District and 71.43% (5/7) NTM/HIV were male with mean age of 43.48 ± 15.06 years.

### NTM disease associated factors

In our univariate analysis, only fever (defined as body temperature of 38 °C or above) and cough were significantly associated with NTM disease (OR 0.41 (CI 0.21–0.81) and OR 0.8 (CI 0.04–0.17), respectively). Our analysis also found that HIV-positive patients had non-significant, but greater odds of having NTM disease compared with those without HIV (OR = 1.44; CI 0.59–3.50; *P* < 0.415).

Multivariate analyses found significant associations between not having cough and lower body weight with NTM disease (OR 0.05 (CI 0.02–0.13) and OR 0.32 (CI 0.11–0.93); Table 2). Our findings suggest that patients suspected of TB who are not coughing and who weigh <55 kg have a great probability of having NTM disease instead of TB.

**Table 2.** Risk factors associated with NTM infections, from 2006 to 2013 in Bamako, Mali

Variables	Non-adjusted (univariate analysis)		Adjusted (multivariate analysis)	
	OR	95% CI	OR	95% CI
Sex				
Male	1	–	1	–
Female	0.78	0.34–1.76	0.54	0.14–1.97
Age range (years)				
(14–30)	1	–	1	–
(30–45)	2.86	1.15–7.12	2.24	0.60–8.39
(45–60)	3.24	1.17–8.98	3.11	0.71–13.51
(60–75)	6.13	1.55–24.24	5.59	0.74–25.12
>75	8.42	1.31–54.17	6.01	0.44–81.51
Body weight (kg)				
<55	1	–	1	–
>55	0.53	0.26–1.09	0.32	0.11–0.93
Fever (°C)				
<37	1	–	1	–
>37	0.41	0.21–0.81	0.69	0.20–2.33
Rails crackling				
No	1	–	1	–
Yes	0.73	0.30–1.72	0.54	0.15–1.93
Cough				
No	1	–	1	–
Yes	0.08	0.04–0.17	0.04	0.01–0.13

OR, odds ratio; 95% CI, 95% confidence interval.

## Discussion

In this study, we determined the prevalence of NTM among individuals suspected of having TB to be 9.3% (41/439) and 12.34% (41/332) among culture positives over the 7 years’ study period at the PGUTH. Conversely, the prevalence of *M. tuberculosis* have NTM and NTM target therapies. With testing of sputum for NTM, these patients would have received several months of unnecessary, expensive and toxic medications targeting TB. Our findings also highlight the need for better diagnostic tools for TB in low- and middle-income countries where NTM prevalence is potentially increasing [8].

Similar findings have been reported by Zida *et al.* in Burkina Faso in 2014 [20] and Maiga *et al.* in Mali in 2012 [4] with NTM prevalence of 11% and 12% in TB suspected cases, respectively.

The age range of those most affected by NTM was 30–40 years and males were more likely to have NTM disease. Similarly, a study by Gerogianni *et al.* [21] in Central Greece found that 67% of NTM-infected individuals were men. We have also found that NTM infection was not driven by HIV infection in Mali with only 17.07% (7/41) of co-infection, although, our data suggest that HIV-infected patients are potentially more vulnerable than others. Another report in Kenya found that 46.7% of NTM disease cases were co-infected with HIV [22]. This difference is likely explained by the fact that HIV is at least six times more prevalent in the general population of Kenya than Mali (nearly 6% and 1%, respectively). Importantly, NTM disease appears to have a greater association with HIV infection in high-prevalence HIV regions, but in low-prevalence HIV areas, such as Mali, NTM remains highly present and possibly driven by other as yet unknown factors. Those factors could be genetics, environment and/or other immunodeficiency disorders. Notably, we found in our study that the absence of cough (with the presence of other symptoms suggestive of TB) and a body weight of <55 kg were associated with diagnosis of NTM disease in those suspected of having TB.

In our study, 50% of NTM cases were due to *M. avium*, which is similar to reports from France in 2004 with 47.7% *M. avium* among NTM cases [5, 22–24]. *M. avium* and many NTM are present in water and often resistant to regular dosing of chloride in public drinking water [25]. Other important NTM reservoirs/sources include soil, dust and bathrooms [8].

This study had several limitations. First, asymptomatic burden of NTM disease could not be assessed due to the inclusion criteria. Second, our study was cross-sectional and without temporality we were unable to assess causality and can only determine associations. Finally, our study may lack generalisability given we utilised a recruitment centre in Bamako with clinically challenging suspected TB cases and those failing TB therapy. Despite these limitations, we consider the impacts of these limitations are minimal on the data interpretations.

## Conclusion and recommendation

Our study was one of the first to assess the burden and risk factors of NTM disease in a TB endemic region of West Africa with low HIV seroprevalence. The prevalence of NTM disease was relatively high in Mali and requires better diagnostic tools to distinguish NTM from TB in this setting. Unlike other places, HIV was not a driving factor and further longitudinal studies are needed to determine the causal factors for the high prevalence of NTM disease we observed. Our data also reinforce that clinical symptoms and sputum smear are not sufficient to effectively diagnose and manage TB-suspected patients.
